# Neuroprotective effect of wormwood against lead exposure

**DOI:** 10.4103/0974-2700.76834

**Published:** 2011

**Authors:** O Kharoubi, M Slimani, A Aoues

**Affiliations:** Department of Biology, Faculty of Science, Laboratory of Biochemistry, University of Es-Senia, Oran, Algeria

**Keywords:** Acetylcholinesterase, behavioral test, brain region, lead acetate, lipid peroxidation, monoamine oxidase

## Abstract

**Background:**

Lead poisoning is a potential factor in brain damage, neurochemical dysfunction and severe behavioral problems. Considering this effect, our study was carried out to investigate the effects of wormwood to restore enzymes activities, lipid peroxidation and behavioral changes induced by lead.

**Methods:**

Thirty Wistar rats were divided into five groups (*n* = 6 in each group): three groups exposed to 750 ppm of lead acetate in the drinking water for 11 weeks and two groups as control. Aqueous wormwood extract (200 mg/kg body weight) was administrated to intoxicated (Pb(-)+A.AB) and control groups (A.AB) for four supplemental weeks. Activities of acetylcholinesterase (AchE), monoamine oxidase (MAO) and thiobarbituric acid-reactive substances (TBARS) level were determined in the hypothalamus, hippocampus, cortex and striatum of male rats and the grooming and locomotors activity were defined in all groups.

**Results:**

The intoxicated group (Pb) has a significantly increased TBARS value compared with the control in all regions (*P* < 0.05) and, after treatment with the wormwood extract, a significant reduction was noted. The enzyme activity decreased significantly (*P* < 0.05) in the Pb group compared with the control, essentially for the hippocampus (AchE: -57%, MAO: -41%) and the striatum (AchE: -43%, MAO: -51%). After wormwood extract administration, the AchE and MAO activity were significantly increased in all brain regions compared with the Pb group (*P* < 0.05). The behavioral test (locomotors and grooming test) indicates a significant hyperactivity in the Pb group compared with the control group. After treatment with wormwood extract, the Pb(-)+A.Ab indicates a lower activity compared with Pb.

**Conclusion:**

These data suggest that wormwood extract may play a very useful role in reduction of the neurotoxicological damage induced by lead.

## INTRODUCTION

Lead (Pb) is a highly neurotoxic agent that particularly affects the developing central nervous system. Lead poisoning is a potential factor in brain damage, mental impairment and severe behavioral abnormalities, neuromuscular weakness, decreased hearing, impaired cognitive functions in experimental animals and coma.[1,2,3] Oxidative stress, oxidative damage to cellular components and activation of the oxidant-sensitive transcription factor could, in part, underlie some of the toxic effects of Pb. The deleterious effects of Pb can involve both reactive oxygen species (ROS) and reactive nitrogen species.

Experimental evidence suggests that cellular damage mediated by free radicals can be involved in the pathology associated with Pb intoxication.[3] In fact, the cerebral damage induced by lead occurs preferentially in the cerebral cortex, cerebellum and hippocampus.[4,5] The cognitive functions are localized in the cerebral cortex while the cerebellum regulates the execution of driving movements, whereas the hippocampus area is the site of memory storage and was implicated in behavioral comportment. Consequently, these anatomical sites are crucial by modulating the emotive answer, memory and behavior, and exposure of the young brain under development to lead can compromise a variety of neurotransmitter systems.[2,4,5]

Recently, the clinical importance of herbal drugs has received considerable attention. As many synthetic antioxidants have been shown to have one or the other side-effects,[6] there has been an upsurge of interest in the therapeutic potential of medicinal plants as antioxidants in reducing free radical-induced tissue injury.[7,8] Numerous plant products have been shown to have an antioxidant activity and the antioxidant vitamins, flavonoids and polyphenolic compounds of plant origin have been extensively investigated as scavengers of free radicals and inhibitors of lipid peroxidation.[9]

Excessive production of radical species plays an important role in neuronal pathology resulting from excitotoxic insults and therefore one plausible neuroprotective mechanism of bioflavonoid is partly relevant to their metal chelating and antioxidant properties. Bioflavonoids are claimed to exert antimutagenic, neurotrophic and xenobiotic ameliorating and membrane molecular stabilizing effects.[10]

Wormwood (*Artemisia absinthium* L.) has a high content of nutrients and phytochemicals such as total phenolic compounds and total flavonoids, suggesting that these compounds contribute to the antioxidative activity.[11] Phenolic substances such as flavonols, cinnamic acids, coumarins and caffeic acids or chlorogenic acids are believed to have antioxidant properties that may play an important role in protecting cells and any organ from oxidative degeneration.[12,13] However, no study has reported the effects of *Artemisia absinthium* L. on lead-induced neurotoxicity. The deficits in learning and memory in Pb-exposed rodents are accompanied by damage to neurons and changes in some neurotransmitters, such as the cholinergic and catecholamine neurotransmitter system are involved.[14,15] In this study, we used behavioral and neurochemical experiments to determine the protective effects of wormwood against the neurotoxicity induced by lead.

## METHODS

### Preparation of wormwood plant extracts (A.Ab)

Whole plants of *Artemisia absinthium* L. were collected from Mecheria, Algeria, in the month of May. The plant was identified and authenticated at the Herbarium of Botany Directorate in Es-Senia (Oran) University. Five hundred grams of whole wormwood plants were extracted with 1.5 L of distilled water by the method of continuous hot extraction at 60°C twice for 30 min and the filtrate was lyophilized. The residue collected (yield 75 g) was stored at -20°C. When needed, the extract was dissolved in distilled water and used in the investigation.

### Animals and tissue preparation

In the experiment, a total of 30 male Wistar rats (18 intoxicated rats, 12 normal rats) were used. The rats were housed five per cage and had free access to food and water, except during testing. They were exposed to a 14–10-h light-dark cycle and the room temperature was controlled at 23 ± 2°C. Animals were first exposed to Pb at the age of 2 weeks, when they weighed 40 ± 6 g.

Experiments were performed during 15 weeks. The 30 Wistar rats were divided into five groups according to:

In the first period:

- *Control*: Rats received water during 11 weeks.
- *Pb group*: Rats exposed to Pb (750 ppm, in the form of Pb acetate in their drinking water *ad libitum*) for 11 weeks.

In the second period:

- *A.Ab group*: After stopped intoxication, the control group received A.Ab extracts at the dose of 200 mg/kg in drinking water *ad libitum* for 4 weeks.
- *Pb(-) group*: After 11 weeks, intoxication by lead was stopped and the rats received water for four additional weeks.
- *Pb(-)+A.Ab groups*: After intoxication, rats received A.Ab extract at the dose of 200 mg/kg in their drinking water *ad libitum* for 4 weeks.

Animals were sacrificed by cervical decapitation under pentobarbital sodium anesthesia (60 mg/kg). The brain was removed, washed with normal saline and all the extraneous materials were removed before weighing. The brain was kept at ice-cooled conditions all the time. The brain was dissected using the method of Glowinski and Iversen[16] into four regions of interest: hypothalamus, hippocampus, cerebral cortex and striatum. Due to the small amount of tissue, tissue of three littermates was pooled.

### Brain cytosolic and mitochondrial fractions

The rat brain tissue was minced and homogenized in 500 μl of buffer A (20 mM HEPES, pH 7.5, 50 mM KCl, 1 mM EDTA, 2 mM MgCl_2_, 220 mM mannitol, 68 mM sucrose, 1 mM leupeptin, 5 μg/ml pepstatin A, 5 μg/ml aprotonin, 0.5 mM PMSF). The homogenate was subjected to differential centrifugation to collect the supernatant (cytosolic fractions) and the pellets (enriched mitochondria fractions). The cytosolic fractions were frozen at -70°C until further analysis. Pellets containing mitochondria were treated with the lysis buffer (1X PBS, 1% NP-40, 0.5% sodium deoxycholate, 0.1% SDS, 250 mM sucrose, 20 mM Tris HCl, pH 7.4, 1 mM DTT and protease inhibitor) and were incubated on ice for 20 min. The lysate was centrifuged at 10,000 g at 30 min at 4°C and the resulting supernatant was kept as the solubilized mitochondrial-enriched fractions and stored at –70°C until further use.

### Estimation of lipid peroxidation

Lipid peroxidation in the brain was estimated colorimetrically by thiobarbituric acid reactive substances (TBARS) by the method of Niehius and Samuelsson.[17] In brief, 0.1 ml of tissue homogenate (Tris-HCl buffer, pH 7.5) was treated with 2 ml of (1:1:1 ratio) TBA-TCA-HCl reagent (thiobarbituric acid 0.37%, 0.25 N HCl and 15% TCA) and placed in a water bath for 15 min and cooled. The absorbance of the clear supernatant was measured against the reference blank at 535 nm.

### Estimation of monoamine oxidase and acetylcholinesterase activity in the brain

The activity of estimation of monoamine oxidase (MAO) was estimated by the method of Green and Haughton.[18] The assay mixture containing 1.0 ml of semicarbazide hydrochloride (0.05 M, pH 7.4), 1.6 ml of phosphate buffer (0.2 M, pH 7.4) and 0.4 ml of mitochondrial fraction was incubated for 20 min at 37°C in a water bath with a shaking device. The reaction was started by adding 0.5 ml of tyramine hydrochloride (0.1 M, pH 7.4) and, after 30 min of incubation, the reaction was stopped by adding 1.0 ml of 0.5 N acetic acid and kept in a boiling water bath for 30 min. The contents were centrifuged for 10 min at 1000× g and, to 2.0 ml of the supernatant, 2.0 ml of 2,4-dinitriphenylhydrazine (0.5 mg/ml in 2N HCl) was added. After keeping at room temperature for 15 min, 5 ml of benzene was added. The tubes were vortexed and the aqueous layer was discarded. The benzene layer was washed with 4 ml of distilled water followed by the addition of 4 ml of 0.1N NaOH solution and the contents of the tubes were mixed thoroughly. The benzene layer was discarded and the NaOH layer was allowed to stand at room temperature for 1 h. The absorbance of the samples was measured at 425 nm. The activity of MAO was calculated using a molar extinction coefficient of 9,500 and expressed as micromoles of *p*-hydroxy phenyl acetaldehyde formed per milligram of protein.

The activity of acetylcholinesterase (AchE) was estimated by the Ellman *et al*.[19] method. This method is based on the measurement of the rate of thiocholine production in the hydrolysis of the substrate acetylcholine. Thiocholine, when reacting with dithiobisnitrobenzoic acid (DTNB), produces a yellow color, which can be measured photometrically. In the reaction mixture, 0.4 ml of synaptosome fractions (average protein content, 0.2–0.4 mg/ml) and 2.6 ml of 0.1 M phosphate buffer, pH 8.0 (containing 0.749 g KH_2_PO_4_, 16.820 g Na_2_HPO_4_2H_2_O and 1,000 ml water) were incubated for 30 min at 37°C under continuous stirring. The samples were moved into photometer cells and 100 μl 5.5-dithiobis-2-nitrobenzoic acid (DTNB) was added. Twenty microliters of the substrate and 0.075 M acetylcholine iodide were added into the photometer cells. The absorbance was measured at 412 nm after 1 min and 5 min. The enzyme activity is expressed as μmol of substrate hydrolyzed/min/mg of protein.

### Neurobehavioral studies

Locomotors activity was evaluated in the open-field test. The open-field behavior of rats was assessed in a box measuring 90 cm × 90 cm × 30 cm, subdivided into 19 equal squares by black lines. Immediately after this, the rat was placed in the center of the open-field, the movements of the rat were scored and the grooming activity (scratching, fur licking, nose washing) was recorded during the 5-min session for 30 min.

### Statistics

The mean ± SEM values were calculated for each group to determine the significance of the intergroup difference. Each parameter was analyzed separately using the one-way analysis of variance (ANOVA) test. To determine the difference between the groups, Student’s “t”-test was used. *P*-values <0.05 were considered to be significant.

## RESULTS

**Figure 1:**
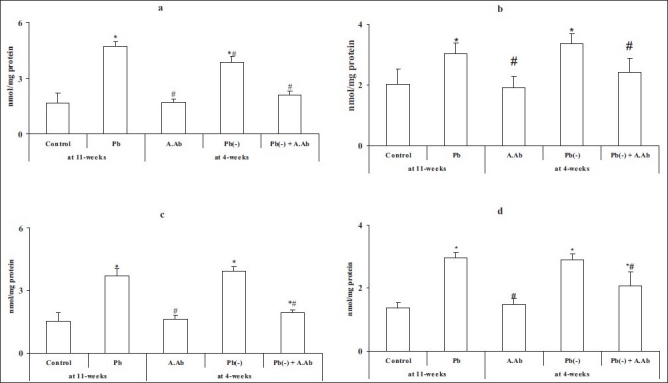
Brain thiobarbituric acid-reactive substances levels in all groups treated and untreated groups with wormwood extract. (a) Hippocampus, (b) striatum, (c) cortex and (d) hypothalamus. Values are mean ± SE (*n* = 6). *P <0.05, Pb group, A.Ab group, Pb(-) group and Pb(-) +A.Ab group were compared vs. control. #*P* <0.05, A.Ab group, Pb(-) group and Pb(-)+A.Ab group are compared vs. Pb group (Student’s “*t*”-test)

A significant increased in blood and urinary lead concentration (respectively, PbB and PbU) was noted between the Pb group (PbU = 6.94 ± 1.7 μg/day, PbB = 55.62 ± 6.30 μg/dl) compared with the control (*P* < 0.05). After stopped intoxication and treatment by wormwood, the level of lead in the Pb(-)+A.Ab group was significantly decreased compared with the Pb(-) group by -46.2% in PbB. The TBARS levels are significantly increased in the Pb group compared with the control group by +182%, +50%, 142% and 114% in the hippocampus, striatum, cortex and hypothalamus, respectively. After wormwood extract administration, a significant reduction (*P* < 0.05) was noted in Pb(-)+A.Ab compared with Pb(-); however, this remains increased compared with the control by +25%, +19%, +28%, +49% and +40% in all regions previously cited [Figure 1].

**Figure 2:**
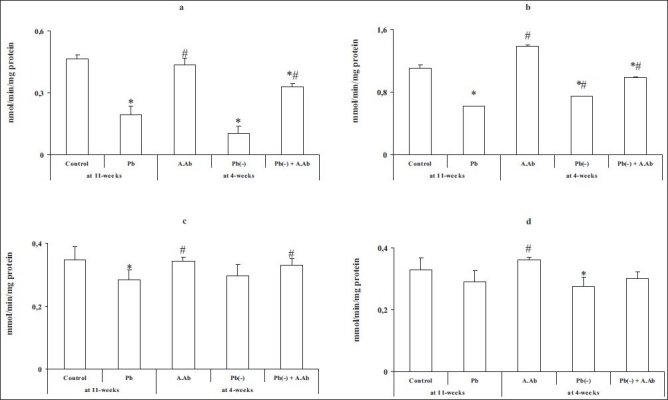
Brain acetylcholinesterase activity in all treated and untreated groups by wormwood extract. (a) Hippocampus, (b) striatum, (c) cortex and (d) hypothalamus. Values are mean ± SE (*n* = 6).*P <0.05, Pb group, A.Ab group, Pb(-) group and Pb(-) +A.Ab group were compared vs. control. #*P* <0.05, A.Ab group, Pb(-) group and Pb(-)+A.Ab group are compared vs. Pb group (Student’s “*>t*”-test)

The activity of AchE was significantly reduced in all brain regions in the intoxicated group vs. the control group after 11 weeks of intoxication (*P* < 0.05), and by -57% in the hippocampus, -43% in the striatum, -18% in the cortex and -11% in the hypothalamus, respectively. After 4 weeks of stopped intoxication, a maximum reduced activity was noted in the hippocampus of the Pb(-) group (-77%) (*P* < 0.05). Administration of wormwood extract indicates a clear significant improvement in the Pb(-)+A.Ab group compared with the Pb group (hippocampus +41%; striatum +37%; cortex +10% and no difference in hypothalamus, respectively) [Figure 2].

**Figure 3:**
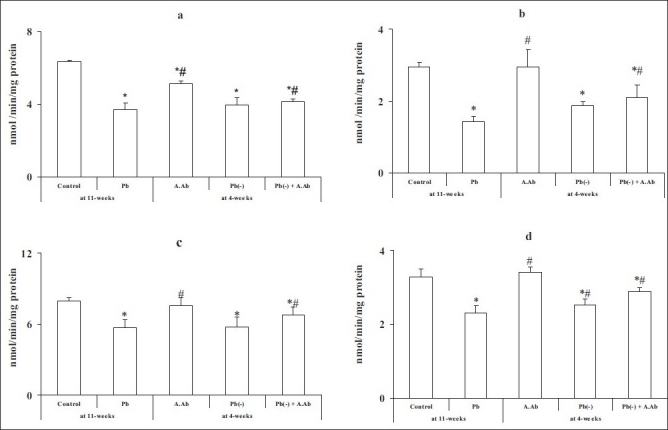
Brain monoamine oxidase activity in all treated and untreated groups by wormwood extract. (a) Hippocampus, (b) striatum, (c) cortex and (d) hypothalamus. Values are mean ± SE (*n* = 6).*P <0.05, Pb group, A.Ab group, Pb(-) group and Pb(-) +A.Ab group were compared vs. control. #*P* <0.05, A.Ab group, Pb(-) group and Pb(-)+A.Ab group are compared vs. Pb group (Student’s “*t*”-test)

We observed a significant decrease (*P* < 0.05) in the MAO activity in different cerebral areas in the Pb group compared with the control group in the hippocampus: -41%; in the striatum: -51%; in the cortex: -28%; and in the hypothalamus: -29%. After treatment with wormwood extract, the Pb(-)+A.Ab group indicated a significant increase (*P* < 0.05) in MAO activity in all brain regions compared with the Pb group, by +10%, +47%, +18% and +24% in the hippocampus, striatum, cortex and hypothalamus, respectively [Figure 3].

**Figure 4:**
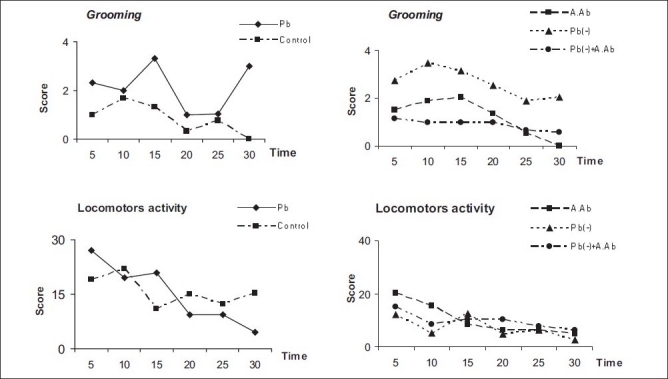
Locomotors and grooming test in all treated and untreated groups

Chronic exposures to lead significantly increase the total general behavior, which included locomotor activity (control 15 ± 2.1 vs. Pb group 21 ± 2.8) and grooming (control 0.9 ± 0.4 vs. Pb group 1.8 ± 0.5). After aqueous wormwood extract administration for 4 weeks, a significant difference score was observed (*P* < 0.05) between the Pb(-)+A.Ab and the Pb group in all tests, by -24.7% in the locomotor activity and -51.3% in the grooming test [Figure 4].

## DISCUSSION

In this study, the effects of aqueous wormwood extract on lead-induced locomotors and grooming impairment and changes in some enzyme activities in different regions of the brain and lipid peroxidation were investigated. The prevention of lead-induced neurotoxic injury by wormwood extract is reported here for the first time. Exposure to lead during early development has been implicated in lasting behavioral abnormalities and cognitive deficits in experimental animals.[3,20] Besides, the present work showed that administration of lead to rats for 11 weeks induced locomotor hyperactivity. Other studies demonstrated the same results in rats treated with lead during the post-natal period.[21]

The hyperactivity found in the present study could be explained by the lead effect on the dopaminergic system and glutamatergic transmission on the level of NMDA receptor (N-métyl-D-aspartate).[14]

It is demonstrated that metal accumulation is associated with high levels of lipid peroxidation in different regions of the brain, such as hippocampus and cerebellum.[22] The vulnerability of neuronal membrane oxidative stress and cellular peroxidation induced by lead is due to the presence of a relatively high concentration of fatty acids that are readily oxidizable. In addition, production of ROS and alteration of homeostasis *in vivo* may be major factors in the severity of lead poisoning.

Pb-exposed rats are consistent with dysfunction of cholinergic innervations.[23] The involvement of the cholinergic system has been implicated by the observations that early lead exposure results in a significant reduction in high-affinity choline uptake in mouse forebrain synaptosomes[24] and a depressed acetylcholine turnover in rat brain.[25] However, these changes in the cholinergic ways could be associated with the peroxydative damage caused on the neuronal membrane.[26]

We observed that administration of lead acetate significantly decreased the MAO activity in various cerebral areas. The work undertaken by Devi *et al*.[5] showed that lead administration modifies the aminergic system by reducting the activity of mitochondrial MAO and tyrosin hydroxylase. The effects observed during exposure to the high Pb levels on MAO and catecholamines at the cerebral level are not a direct consequence of the intoxication by lead but a resultant of the inhibiting effect of the cholinergic system.[27] The reduction in the activity of MAOs in the various cerebral areas, during the exposure to lead, can be due to the cellular damage[28] and with the high affinity of Pb to sulfhydryl group of these enzymes.[29]

In addition, we observed that administration of the aqueous extract of wormwood has a clear improvement in the various behavioral tests compared with rats exposed to lead. In the same way, we recorded that administration of plant extract after stopped poison induced a re-establishment of the enzymatic activities (AchE and MAO) and significantly reduced the TBARS values in the various cerebral structures compared with the Pb groups. The prophylactic effectiveness of this extract can be allotted to its antioxidant action and/or its chelating capacity due primarily to the action of sulfhydryl groups. These results agree with the fact that the natural compounds rich in antioxidants involve a considerable improvement in the enzyme activity and reduce oxidative stress,[30] which plays a significant role in the toxicity inversion of lead by forming inert complexes and inhibiting its toxicity on the dopaminergique neurons.[31]

## CONCLUSION

In conclusion, lead exposure induced a significant behavioral alteration as well as neurochemical alteration in different brain regions in rats exposed to Pb. Moreover, *Artemisia absinthium* L. prevents neurotoxicity induced by lead by decreasing the lipid peroxidation, modifying locomotor behaviors and grooming and restoring enzyme activities involved in the regulation of some neurotransmitters.
